# DeepFusion-HSK: a multimodal deep learning for subtype classification of herpes simplex keratitis with multicenter validation

**DOI:** 10.3389/fcell.2026.1978214

**Published:** 2026-10-19

**Authors:** Zuhui Zhang, Tao Lv, Jiahao Tang, Kunhui Xu, Fen-Fen Li, Xinxin Yu, Chenchen Wang, Shuangqing Wu, Ya-na Fu, Jie-Wei Jiang, Fan Lu, Qi Dai

**Affiliations:** 1 National Clinical Research Center for Ocular Diseases, Eye Hospital, Cixi Biomedical Research Institute, Wenzhou Medical University, Wenzhou, China; 2 School of Electronic Engineering, Xi’an University of Posts and Telecommunications, Xi’an, China

**Keywords:** herpes simplex keratitis, multicenter validation, multimodal deep learning, slit-lamp imaging, subtype classification

## Abstract

**Introduction:**

To develop and evaluate a multimodal deep learning (DL) model for subtype classification of viral keratitis.

**Methods:**

This retrospective multicenter study included 3,501 slit-lamp images (807 eyes) as the internal development set and two external validation cohorts (2,957 images total). DeepFusion-HSK integrates three complementary illumination modalities—diffuse light imaging (DLI), slit-beam imaging (SBI), and fluorescein staining imaging (FSI)—through a novel fusion architecture. The framework uses a tri-way higher-order interaction layer to capture cross-modal pathological signatures, dual-task learning with auxiliary heads to prevent modality-specific feature degradation, and a dynamic gating mechanism that uses high-confidence coarse priors to calibrate fine-grained predictions and mitigate softmax probability spillover. To simulate real-world data deficiency, we evaluated model resilience under complete FSI dropout.

**Results:**

On the internal test set, DeepFusion-HSK achieved an accuracy of 85.1% (95% CI: 78.5%–90.0%) and AUCs of 0.947–0.949 for discriminating epithelial herpes simplex keratitis (E-HSK) and non-epithelial herpes simplex keratitis (NE-HSK), significantly outperforming Swin-Transformer, EfficientNetV2, and ResNet50. External validation confirmed robust generalization, with accuracies of 81.7% on Dataset I and 78.2% on Dataset II, demonstrating clinical resilience. Ablation studies confirmed the hierarchical synergy of multimodal inputs: the best single modality (FSI) yielded 78.5% accuracy, the optimal dual-modality combination (DLI + FSI) reached 81.7%, and full tri-modal fusion improved performance to 85.1%, validating that integrated global morphology, depth contour, and superficial ulcer signatures are essential for robust HSK subtyping.

**Discussion:**

DeepFusion-HSK offers a robust, generalizable, and clinically deployable solution for therapeutic HSK subtyping. Its resilience to missing imaging modalities makes it well suited for heterogeneous real-world ophthalmic practice, providing a decision-support tool to reduce vision-threatening misdiagnoses and guide therapy.

## Introduction

Herpes simplex keratitis (HSK) results from infection with herpes simplex virus type 1 (HSV-1). Primary infection can involve ocular or non-ocular sites, after which latency is established primarily in the trigeminal ganglion but also in the cornea. The initial ocular infection may be mild. Visual morbidity results from recurrent keratitis, which leads to corneal scarring, thinning, and neovascularization (Kaye and Choudhary, 2006). HSK is one of the leading causes of blindness worldwide and places a significant burden on public health systems. The global incidence of HSK is roughly 1.5 million, including 40,000 new cases of severe monocular visual impairment or blindness each year (Farooq and Shukla, 2012). Results from epidemiological surveys conducted in Chinese populations indicate that the incidence of HSK is approximately 65–110 per 100,000 population (Cao et al., 2014; Song et al., 2014). Epithelial dendritic lesions represented the most frequent type of recurrent keratitis (56.3%), followed by herpes stromal keratitis (29.5%), and geographic epithelial lesions (9.8%) (Labetoulle et al., 2005). HSK lesions are immunoinflammatory and can recur throughout life, often causing progressive corneal scarring and visual impairment. Current treatment involves broad local immunosuppression with topical steroids plus antiviral coverage (Rowe et al., 2013). Therefore, precise subtyping of HSK is therapeutically imperative.

There are four major categories of HSK (Holland and Schwartz, 1999): (1) epithelial herpes simplex keratitis (E-HSK), comprising corneal vesicles, dendritic ulcers, geographic ulcers, and marginal ulcers; (2) neurotrophic keratopathy, including punctate epithelial erosions and neurotrophic ulcers; (3) stromal keratitis, subdivided into necrotizing and immune stromal keratitis; and (4) endotheliitis, with three clinical presentations: disciform, diffuse, and linear. Clinically, E-HSK typically presents as characteristic epithelial ulcers requiring immediate and aggressive antiviral treatment. In contrast, non-epithelial herpes simplex keratitis (NE-HSK) typically involves viral endotheliitis or deep stromal inflammation, requiring careful modulation of the host immune response with topical corticosteroids alongside antiviral prophylaxis (Farooq and Shukla, 2012; Labetoulle et al., 2005; Labib and Chigbu, 2022). Misdiagnosing E-HSK as NE-HSK or other corneal abnormalities and subsequently administering corticosteroids can trigger rapid viral replication, catastrophic ulcer progression, and severe visual sequelae (Chodosh, 2020). HSK is frequently misdiagnosed clinically. In developing countries without access to PCR testing, diagnosis relies solely on slit-lamp biomicroscopy; consequently, many HSK cases are misclassified as unexplained corneal opacities or non-infectious keratitis (Farooq and Shukla, 2012). Therefore, a method that can accurately and rapidly identify the cause of HSK is essential to reducing vision loss through timely and effective treatment.

Artificial intelligence (AI) has transformed ophthalmic image analysis. AI-assisted image analysis and automated annotation tools enhance educational resources, reduce learning time, and improve diagnostic accuracy (Yuan et al., 2025). Numerous AI systems have achieved expert-level performance in detecting diabetic retinopathy, glaucoma, age-related macular degeneration, macular edema, neuro-ophthalmologic disorders, and anterior segment diseases (Cao et al., 2025; Feng et al., 2023; Kozma et al., 2025; Liu et al., 2024; Su et al., 2018; Wan et al., 2022; Wang et al., 2022; Wu et al., 2024; Mutua et al., 2025; Shao et al., 2025; Tang et al., 2024). Despite these advances, most AI models for keratitis classification rely on single-modality imaging, typically diffuse slit-lamp images. Such single-modality approaches are information-limited and constrain diagnostic sensitivity and clinical utility in complex real-world settings (Ghosh et al., 2022; Won et al., 2023; Soleimani et al., 2023). More importantly, a substantial gap remains: nearly all prior studies have grouped E-HSK and NE-HSK into a single “viral keratitis” category, overlooking the therapeutic distinction that separates them.

Building on our previous work, which established a multimodal slit-lamp image-based AI system for general infectious keratitis classification (Li et al., 2026), we introduce DeepFusion-HSK, a multimodal deep learning (DL) framework designed to address this subtyping gap. By integrating diffuse light, slit-beam, and fluorescein-stained imaging, the framework overcomes the information constraints of single-modality systems. The clinical goal is to provide ophthalmologists—particularly early-career practitioners—with a robust, interpretable decision-support tool that improves diagnostic accuracy and guides appropriate therapy, thereby preventing vision-threatening outcomes from inappropriate treatment decisions.

## Materials and methods

### Image datasets

This study was approved by the Medical Ethics Committee of the Eye Hospital of Wenzhou Medical University (H2023-049-K-45), and the Chinese Clinical Trial Registry (No. ChiCTR2400086787). The study protocol fully adhered to the Declaration of Helsinki and the Association for Research in Vision and Ophthalmology (ARVO) statement on human subjects. In this study, a total of 3,501 slit-lamp images (3,945 × 2,620 pixels in JPG format) that were consecutively collected from 807 eyes at internal dataset between 2018 and 2025 were employed to develop an AI system. To comprehensively evaluate the generalization capability of DeepFusion-HSK, we employed two independent external datasets from distinct clinical centers: External Dataset I from Aier Guangming Eye Hospital (AGEH) and External Dataset II from The Second Affiliated Hospital, Zhejiang University School of Medicine (SAHZU). External Dataset I, sourced from AGEH, comprised 1780 tri-modal images from 142 patients. External Dataset II, obtained from SAHZU, contained 1,177 images from 131 patients. The overall study design is illustrated in Figure 1.

**FIGURE 1:**
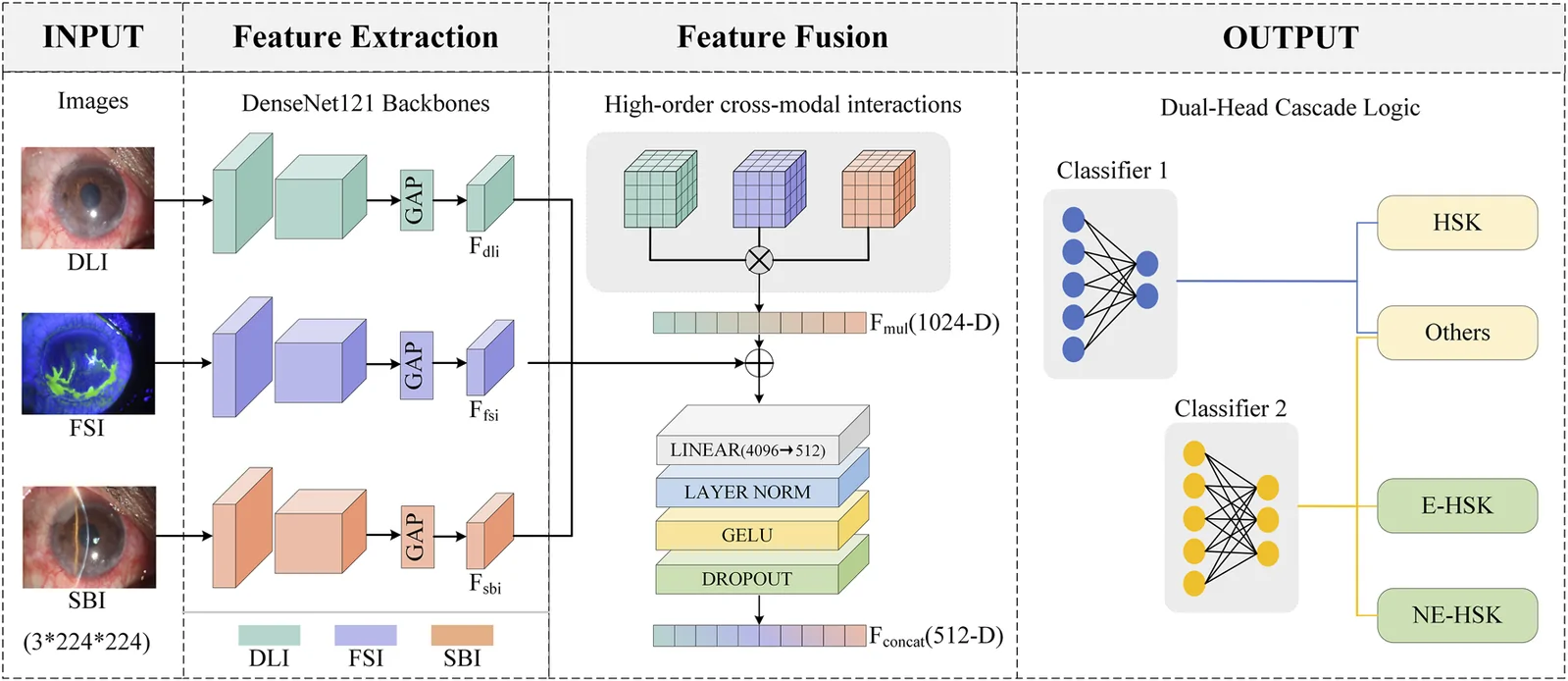
Overall architecture of the DeepFusion-HSK model. The framework processes three slit-lamp illumination modalities (DLI, SBI, and FSI) through modality-specific feature extractors, followed by a fusion module with multiplicative interaction, dual-task classification heads, and a dynamic gating mechanism. E-HSK: epithelial herpes simplex keratitis; NE-HSK: Non-epithelial herpes simplex keratitis; Others: other corneal abnormalities; DLI: diffuse light imaging; SBI: slit-beam imaging; FSI: fluorescein staining imaging.

The reference standard was based on the final documented clinical diagnosis in the medical records, which had been established during routine care according to disease characteristics, clinical history, treatment course, and relevant laboratory findings where applicable. The enrolled patients met one of the following criteria: (1) slit-lamp images obtained with corneal infections in the active stage; (2) patients suspected of having HSK based on their medical histories, clinical manifestations, exclusion of bacterial keratitis (BK), fungal keratitis (FK), and non-infectious corneal diseases (NIK) and effective responses after empirical antiviral therapy; and (3) BK, FK, and NIK were grouped as “others” based on their characteristic clinical features. The NIK category comprised immune-mediated keratitis, toxic keratopathy, and exposure keratopathy. During dataset construction, two ophthalmologists jointly reviewed the medical records and corresponding images in face-to-face sessions to verify eligibility and label consistency according to the predefined diagnostic criteria. This procedure served as a quality-control measure to minimize potential errors that might arise from single-reviewer data collection during retrospective chart review. Cases with uncertain diagnoses, diagnostic ambiguity, mixed infections, or incomplete diagnostic information were excluded. Additional exclusion criteria were a history of ocular trauma or eye surgery and absence of slit-lamp images. Representative image series for each category is shown in Figure 2.

**FIGURE 2:**
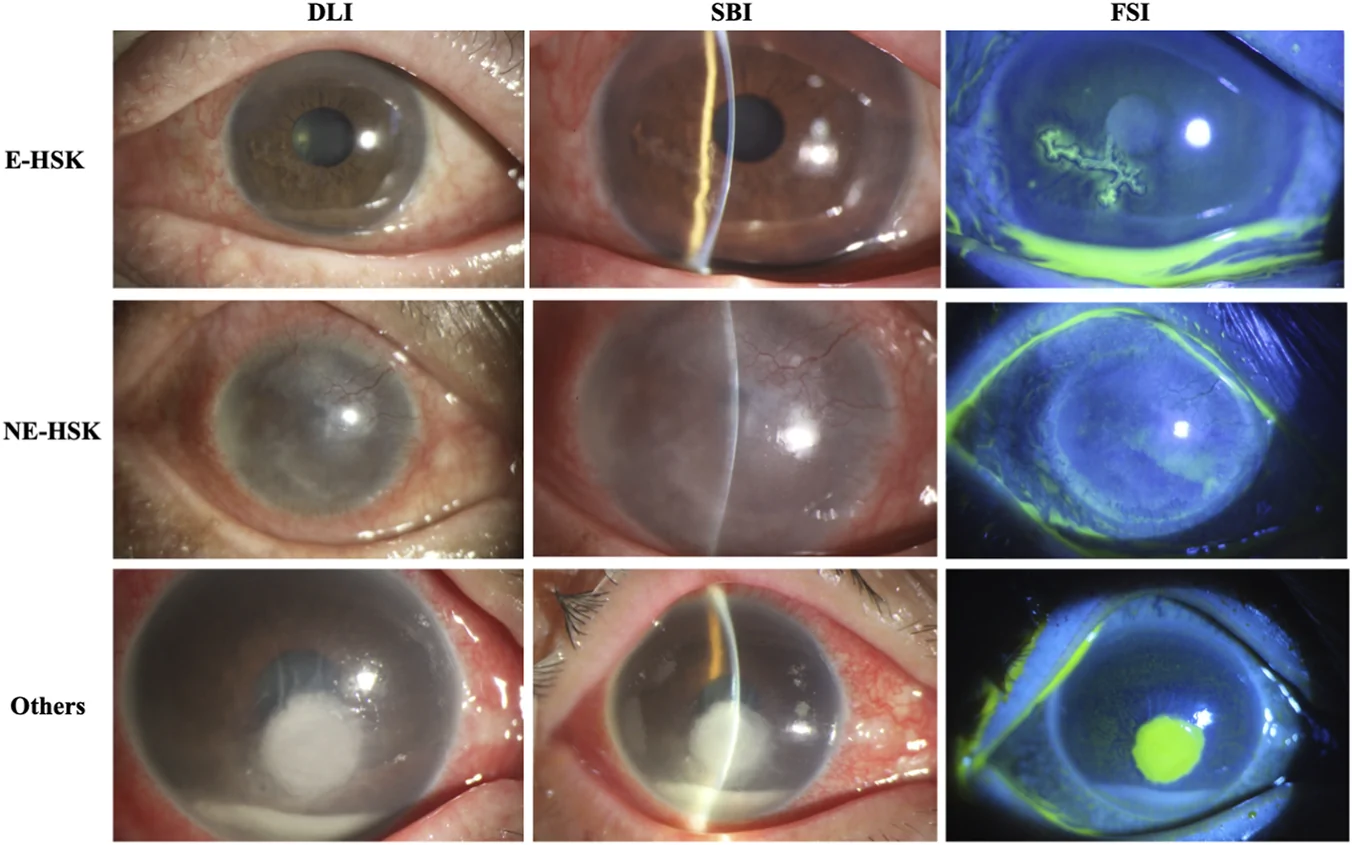
Representative slit-lamp images for each diagnostic category and illumination modality. E-HSK: epithelial herpes simplex keratitis; NE-HSK: Non-epithelial herpes simplex keratitis; Others: other corneal abnormalities; DLI: diffuse light imaging; SBI: slit-beam imaging; FSI: fluorescein staining imaging.

### Image preprocessing

Slit-lamp images were acquired using three digital camera systems (Kanghua DED-3H, Canton Shangbang LS-7, and Shangbang SL-6) under three standardized illumination modalities: slit-beam imaging (SBI), diffuse light imaging (DLI), and fluorescein staining imaging (FSI). To ensure cross-device consistency, a standardized quality-control pipeline, previously detailed in our prior work (Li et al., 2025), was uniformly applied across all modalities. DLI provides uniform anterior-segment illumination, facilitating comprehensive evaluation of surface integrity, irregularities, and pathological changes such as ulceration or inflammation. SBI employs a narrow optical section to delineate corneal fine structures, enabling assessment of transparency, contour, thickness, and depth. FSI, under cobalt-blue light, highlights epithelial defects as green-stained areas, thereby allowing precise identification of corneal injuries and abnormalities.

Owing to the inherent differences between these categories, their images can be considered from different modalities. Directly combining them in a single network for processing may lead to interference between these modalities, as the feature disparities across various modalities make it challenging for the model to effectively capture the relevant features of each modality, thereby limiting the accuracy of the developed model. All images were uniformly resized to 224 × 224 pixels and pixel values were normalized. To prevent overfitting and enhance the model’s generalization ability, image augmentation techniques were employed, including random rotation, horizontal and vertical flipping, and coarse-grained occlusion simulating clinical occlusions, eyelid interference, or strong light reflection artifacts commonly encountered in ophthalmic imaging. The above image processing ensures consistency across all three modalities. Therefore, this study proposes DeepFusion-HSK, an DenseNet designed to integrate imaging data derived from different modalities for classification tasks, including SBI, diffuse light imaging (DLI), and fluorescein staining imaging (FSI). The network uses three independent expert models, each of which is trained to process images acquired from a specific modality.

### Design of DeepFusion-HSK

The development dataset was divided into training, validation, and test sets at a ratio of 7:1.5:1.5. Stratified sampling was performed at the patient level: Each eye was treated as an independent sample, and all images from the same patient were assigned to the same subset. Within each diagnostic category (E-HSK, NE-HSK, and Others), patients were randomly allocated while preserving the original class proportions. This ensures consistent class distributions across subsets and prevents data leakage. Stratified sampling is appropriate here because the three categories differ in prevalence, and consistent representation across subsets is necessary for unbiased performance evaluation. Additionally, all relevant images from the same patient were included in the same partition to completely eliminate the risk of data leakage. In this study, corresponding sections common to the three modalities—DLI, SBI, and FSI—were extracted to form strictly paired tri-modal inputs. Model training utilized the DenseNet121 convolutional neural network architecture, loading DeepIK model weights trained on large-scale, multicenter ophthalmic data as initialization parameters. This incorporated extensive prior knowledge of corneal anatomical structures and pathological features, enabling the model to rapidly focus on key lesion areas and significantly accelerate convergence.

To simulate the comprehensive diagnostic reasoning process of clinical ophthalmologists, who take into account macroscopic anterior segment structures, internal hierarchical structures, and superficial lesion characteristics, this algorithm constructs a three-modal fusion framework specifically designed for multimodal keratitis classification, named DeepFusion-HSK, as shown in Figure 1. This framework processes corresponding images from DLI, SBI, and FSI simultaneously without sharing weights. The feature vectors extracted from the respective modal backbone networks, DenseNet121, are denoted as F_dli_, F_sbi_, and F_fsi_ ∈R^1024^. These three modal vectors are unified through a tri-way higher-order interaction fusion layer, where they are multiplied element-wise to produce F_mul_ see Equation 1. This enables the three modalities to complement each other’s feature information, allowing the model to capture complex cross-modal interactions and reveal synergistic pathological patterns.

$$F_{mul}=F_{dli}⊙F_{sbi}⊙F_{fsi}\in R^{1024}$$ (1)

Next, the three modality-specific feature vectors are concatenated with the multiplicative interaction feature along the feature dimension to form the final feature, denoted as F_concat_ see Equation 2:

$$F_{concat}=Concat\left(F_{dli},F_{sbi},F_{fsi},F_{mul}\right)\in R^{4096}$$ (2)

Here, R^4096^denotes a 4096-dimensional real-valued feature space. The dimensionality is obtained by concatenating four 1024-dimensional feature vectors. Thus, F_concat_ preserves both the modality-specific representations and their multiplicative cross-modal interaction. A lightweight fusion block, utilizing layer normalization and GELU activation, compresses the features into a highly discriminative shared representation, which is then fed into the classifier. The traditional classification head is replaced by a dual-task architecture, where the fused representation is directed to two parallel classifiers: Classifier one aims to distinguish between viral and non-viral infections, providing macro-level prior knowledge; while Classifier two performs fine-grained three-class classification. Furthermore, to enforce feature learning for each modality and prevent feature degradation, independent auxiliary heads are assigned to each modal backbone prior to fusion. DeepFusion-HSK is trained by minimizing a joint cross-entropy loss function see Equation 3, defined as follows:

$$Loss=\alpha \times Loss\_C1+\left(1-\alpha \right)\times Loss\_C2+\beta \times \sum_{m\in M}Loss\_aux\_m$$ (3)

Here, Loss_C1 and Loss_C2 represent the losses of the dual-task classifier; α is the balancing parameter, set to 0.5; β represents the weight of auxiliary supervision, set to 0.3; and M denotes the set {DLI, SBI, FSI}. To address class imbalance and the inherent ambiguity in distinguishing between HSK and others, a cost-sensitive learning method was implemented, assigning class weights of 1.5, 1.0, and 1.0 to the E-HSK, NE-HSK, and others, respectively, in Loss_C2. To further evaluate the effect of the imbalance-handling strategy, we conducted an additional controlled comparison using Focal Loss as an alternative to the class-weighted cross-entropy loss. The class-weighted model achieved better performance than the Focal Loss model in the primary evaluation metrics. The detailed comparison is presented in Supplementary Table S1. During the testing phase, a dual-head gating mechanism is introduced. By using the high-confidence output of Classifier 1 as a prior signal, this mechanism conditionally intercepts and calibrates the output of Classifier 2, effectively mitigating the probability spillover artifacts commonly observed in the standard Softmax function.

### Statistical analysis

To report the performance characteristics of this three-class multimodal fusion model, we calculated accuracy, sensitivity, specificity, F1 score, and the area under the receiver operating characteristic (ROC) curve (AUC) to quantify the model’s performance. Two-sided 95% confidence intervals for accuracy, sensitivity, specificity, and F1 score were determined using a patient-level bootstrap percentile procedure with 1,000 resampling attempts. A confusion matrix was used to present the classification results. The 95% confidence interval for AUC was calculated using the same patient-level bootstrap percentile procedure.

### Implement details

The code for model training and evaluation was developed using Python 3.11.15 and the PyTorch 2.11.0 framework. The hardware environment consists of an RTX 6000 Ada Generation GPU with 48 GB of memory. During training, the maximum number of iterations was set to 150, the batch size to 16, and the weight decay to 5e-3. The initial learning rate was set to 1e-4 and adjusted using a cosine-smoothed annealing scheduler. The Gradient-Sensitive Minimization (SAM) optimizer was combined with the AdamW base optimizer to seek flatter local minima, thereby enhancing generalization capabilities across heterogeneous clinical cases. Additionally, an exponential moving average was applied to the model weights to stabilize training. In each training round, the model weights that achieved the highest F1 score on the validation set were saved and ultimately used for testing on the internal test set.

## Result

### Characteristics of the datasets

The final internal dataset comprises 3,501 images from 807 affected eyes. This includes 723 images of E-HSK, 1,008 images of NE-HSK, and 1770 images of other corneal abnormalities, comprising 474 images from 109 eyes with BK, 603 images from 121 eyes with FK, and 693 images from 175 eyes with NIK. The training, validation, and test sets were divided in a 7:1.5:1.5 ratio. Further information on datasets from the internal test dataset, external test dataset I, and external test dataset II is summarized in Table 1.

**TABLE 1:** Dataset characteristics.

Clinical center	Internal dataset	ExternalI	ExternalII
Location of center	Hangzhou, Zhejiang	Ningbo, Zhejiang	Hangzhou, Zhejiang
Eyes	807	142	131
Total no. of images	3,501	1780	1,177
E-HSK	723	342	49
NE-HSK	1,008	700	356
Others	1770	738	772

E-HSK: epithelial herpes simplex keratitis; NE-HSK: non-epithelial herpes simplex keratitis.

#### Performance of different DL algorithms in the internal test dataset

Using an internal dataset, four DL algorithms, namely ResNet50, EfficientNetV2, Swin-Transformer, and our proposed DeepFusion-HSK,were employed to train models for diagnosing E-HSK, NE-HSK, and others based on three types of slit-lamp images.

The performance of these four algorithms in the internal test dataset is described in Figures 3A,B, which illustrate that the best algorithm is the DeepFusion-HSK. Further information including accuracies, precision, sensitivities, specificities, and F1-scores of these algorithms is shown in Table 2. For the classification of E-HSK, NE-HSK, and other corneal abnormalities, DeepFusion-HSK achieved area under the receiver operating characteristic curve (AUC) values of 0.947 (95% CI: 0.892–0.987), 0.949 (95% CI: 0.904–0.985), 0.940 (95% CI: 0.889–0.979), outperforming other algorithms. DeepFusion-HSK also demonstrated superior performance on other metrics, with an accuracy of 85.1% (95% CI: 78.5–90.0). It was followed by Swin-Transformer, EfficientNetV2, and ResNet50. The F1 scores for the four algorithms were 84.1% (95% CI: 77.0–90.4), 78.5% (95% CI: 70.2–86.1), 75.1% (95% CI: 66.3–83.3), and 46.4% (95% CI: 38.8–54.5). These results indicate that, compared with other algorithms, DeepFusion-HSK performs better in diagnosing the three types of corneal lesions.

**FIGURE 3:**
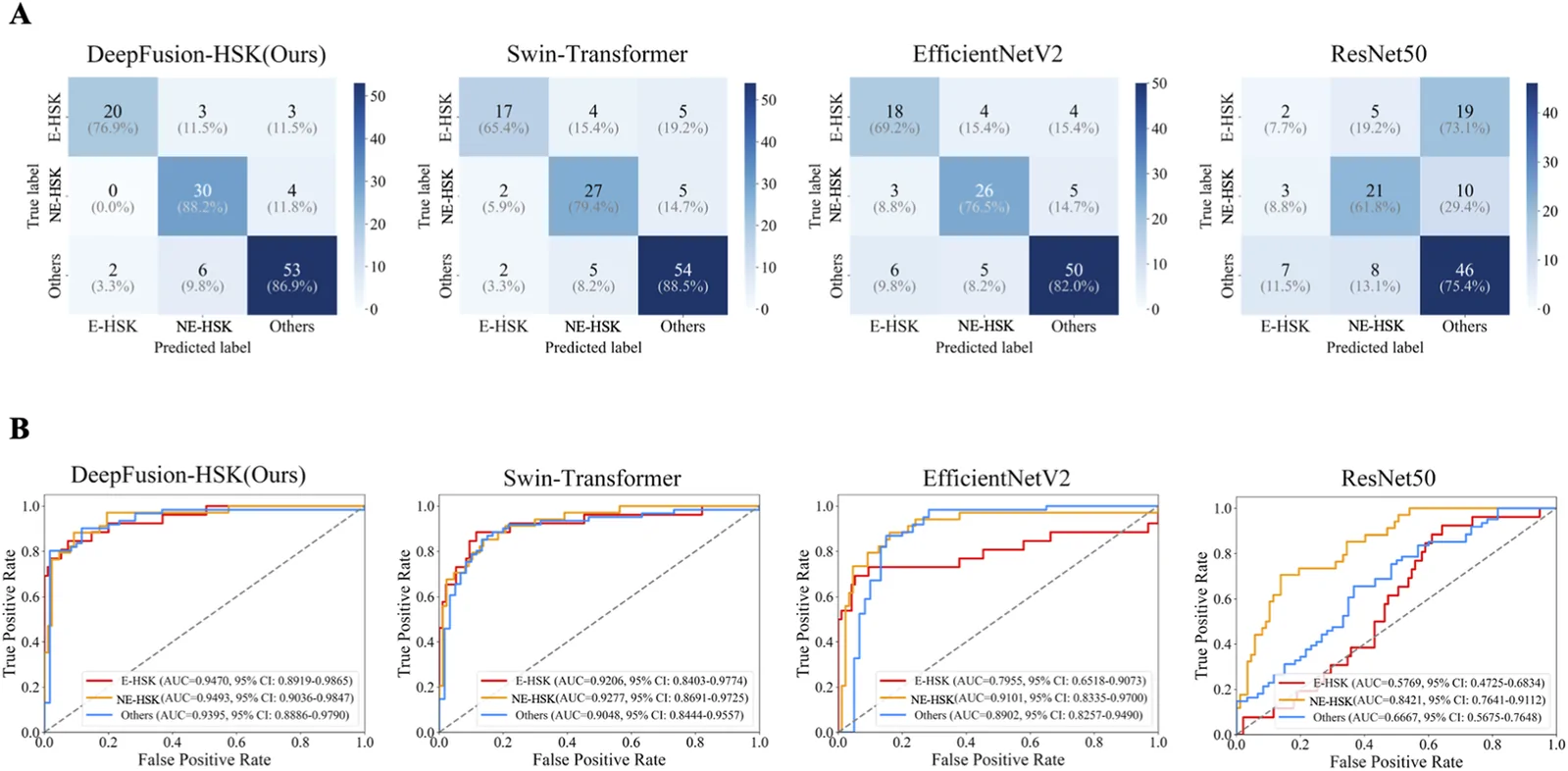
Diagnostic performance of four deep learning algorithms on the internal test set. **(A)** Confusion matrices showing classification results for E-HSK, NE-HSK, and Others by DeepFusion-HSK, Swin-Transformer, EfficientNetV2, and ResNet50. **(B)** Receiver operating characteristic curves for each algorithm, with area under the curve values for the three diagnostic categories. E-HSK: epithelial herpes simplex keratitis; NE-HSK: Non-epithelial herpes simplex keratitis; Others: other corneal abnormalities; DLI: diffuse light imaging; SBI: slit-beam imaging; FSI: fluorescein staining imaging.

**TABLE 2:** Performance comparisons of various prediction models trained and evaluated on the internal dataset.

Model	DeepFusion-HSK	Swin-transformer	EfficientNetV2	ResNet50
Accuracy (%)	85.1 (78.5–90.0)	81.1 (73.6–87.6)	77.6 (70.3–85.1)	57.1 (47.9–66.1)
Precision (%)	85.3 (78.1–91.2)	80.2 (71.4–88.0)	75.1 (66.2–83.2)	46.6 (37.0–57.3)
Sensitivity (%)	83.9 (76.4–90.7)	77.9 (69.6–86.1)	75.8 (67.0–83.9)	48.4 (40.8–51.2)
Specificity (%)	91.9 (88.4–95.1)	89.7 (85.7–93.5)	88.4 (84.4–92.2)	75.5 (70.7–80.2)
F1-score (%)	84.1 (77.0–90.4)	78.5 (70.2–86.1)	75.1 (66.3–83.3)	46.4 (38.8–54.5)

#### Performance of different DL algorithms in the external test dataset

To comprehensively evaluate the generalization capability of our proposed model, we conducted external validation using two independent datasets from distinct clinical centers. Correspondingly, the receiver operating characteristic (ROC) curves and the confusion matrices (Figures 4, 5) of these algorithms in the external datasets indicated that the DeepFusion-HSK algorithm has the best performance in the classification of E-HSK, NE-HSK, and others. Detailed performance metrics for External Dataset I and External Dataset II are presented in Table 3 and Table 4, respectively. On External Dataset I, DeepFusion-HSK attained an accuracy of 81.7% (95% CI: 75.4%–88.0%), outperforming all comparison models. On External Dataset II, a notable challenge arose due to the complete absence of the FSI modality; we therefore replaced it with all-black images and fine-tuned the model on 30 randomly selected patients to adapt to this missing-modality scenario, before testing on the remaining 101 patients. Despite this data deficiency, DeepFusion-HSK still achieved a superior accuracy of 78.2% (95% CI: 70.3%–85.2%), maintaining its leading position among all evaluated algorithms.

**FIGURE 4:**
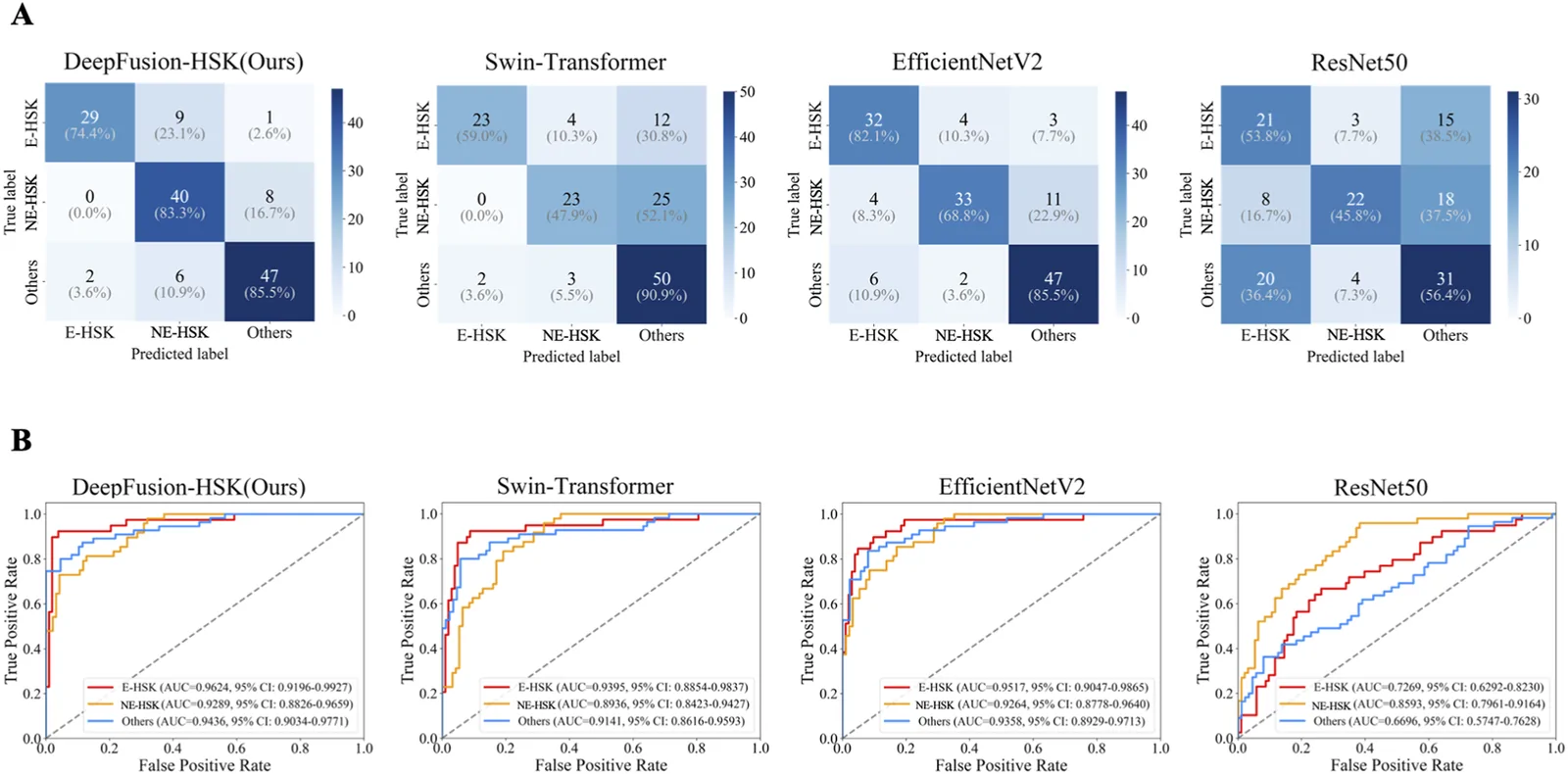
Diagnostic performance of four deep learning algorithms on the external dataset I. **(A)** Confusion matrices showing classification results for E-HSK, NE-HSK, and Others by DeepFusion-HSK, Swin-Transformer, EfficientNetV2, and ResNet50. **(B)** Receiver operating characteristic curves for each algorithm, with area under the curve values for the three diagnostic categories. E-HSK: epithelial herpes simplex keratitis; NE-HSK: Non-epithelial herpes simplex keratitis; Others: other corneal abnormalities; DLI: diffuse light imaging; SBI: slit-beam imaging; FSI: fluorescein staining imaging.

**FIGURE 5:**
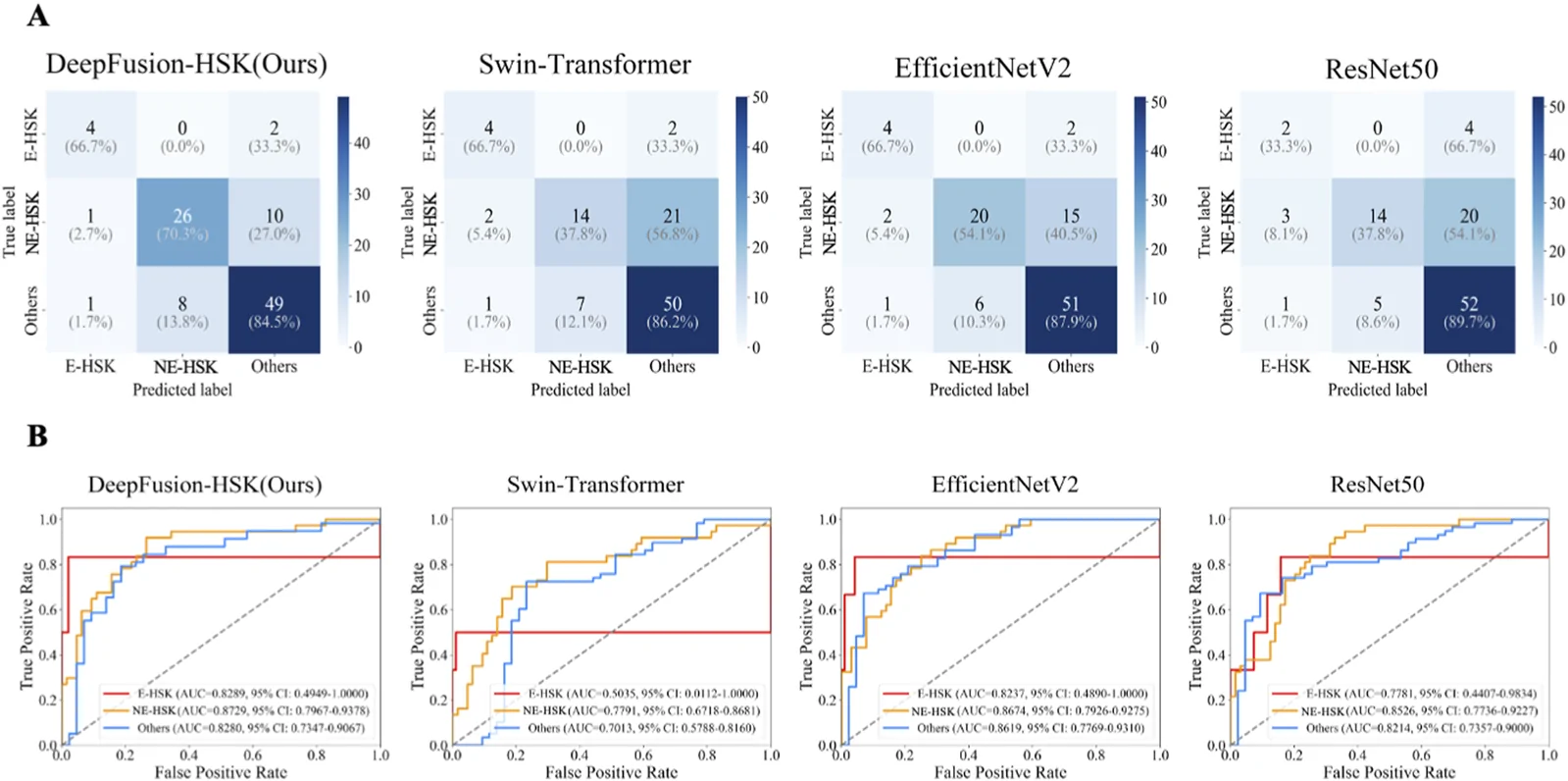
Diagnostic performance of four deep learning algorithms on the external dataset II. **(A)** Confusion matrices showing classification results for E-HSK, NE-HSK, and Others by DeepFusion-HSK, Swin-Transformer, EfficientNetV2, and ResNet50. **(B)** Receiver operating characteristic curves for each algorithm, with area under the curve values for the three diagnostic categories. E-HSK: epithelial herpes simplex keratitis; NE-HSK: Non-epithelial herpes simplex keratitis; Others: other corneal abnormalities; DLI: diffuse light imaging; SBI: slit-beam imaging; FSI: fluorescein staining imaging.

**TABLE 3:** Performance comparisons of various DL models trained and evaluated on the external dataset I.

Model	DeepFusion-HSK	Swin-transformer	EfficientNetV2	ResNet50
Accuracy (%)	81.7 (75.4–88.0)	67.6 (59.9–75.4)	78.9 (71.8–85.2)	52.1 (43.7–60.6)
Precision (%)	83.4 (77.4–89.4)	80.2 (71.4–88.0)	79.3 (72.4–86.0)	55.7 (47.5–63.7)
Sensitivity (%)	81.1 (74.2–87.5)	65.9 (58.2–73.2)	78.9 (72.2–85.4)	52.0 (43.3–60.5)
Specificity (%)	90.6 (87.2–93.9)	82.7 (78.9–86.4)	89.3 (86.0–92.7)	75.8 (71.6–80.0)
F1-score (%)	81.7 (75.0–88.0)	67.1 (58.7–74.5)	78.6 (71.6–85.2)	52.3 (43.4–60.4)

DL: Deep learning.

**TABLE 4:** Performance comparisons of various DL models trained and evaluated on the external dataset II.

Model	DeepFusion-HSK	Swin-transformer	EfficientNetV2	ResNet50
Accuracy (%)	78.2 (70.3–85.2)	67.3 (57.4–76.2)	74.3 (65.4–82.2)	67.3 (58.4–77.2)
Precision (%)	74.5 (57.7–88.0)	65.1 (57.1–79.1)	69.7 (54.2–83.1)	58.5 (43.1–77.8)
Sensitivity (%)	73.8 (57.3–87.6)	63.6 (56.7–77.3)	69.6 (52.0–83.1)	53.6 (49.9–70.3)
Specificity (%)	85.8 (80.3–90.6)	77.5 (72.0–82.8)	82.6 (77.1–87.8)	77.4 (72.0–82.7)
F1-score (%)	74.1 (57.8–85.2)	62.1 (54.7–74.3)	68.7 (52.0–78.7)	53.7 (49.1–68.2)

#### Ablation study

To systematically verify the independent contributions of each imaging modality and rigorously evaluate the effectiveness of the proposed three-modality fusion framework, this study conducted an extensive set of ablation experiments. The experiments covered single-modality (DLI, SBI, FSI), dual-modality (DLI + SBI, DLI + FSI, SBI + FSI), and three-modality (DLI + SBI + FSI) configurations. All compared models shared identical backbone architecture, training hyperparameters, and data splitting strategies to ensure fair comparisons.

As summarized in Table 5 and Figure 6, substantial performance disparities were observed among single-modality models, reflecting the distinct pathological features captured by each imaging technique. Notably, the FSI-based model achieved the highest single-modality accuracy of 78.5% (95% CI: 71.1%–86.0%), owing to its superior ability to highlight corneal epithelial defects and dendritic ulcers through fluorescein staining. In contrast, the SBI-only model yielded the lowest accuracy at 63.6% (95% CI: 55.5%–71.1%), likely due to the noisier and less discriminative features extracted from slit-beam imaging. The DLI-only model, which provides clear overall ocular surface morphology, attained a moderate accuracy of 73.5% (95% CI: 65.3%–81.8%).

**TABLE 5:** Ablation study of modality configurations.

Modality	Accuracy (%)	Precision (%)	Sensitivity (%)	Specificity (%)	F1-score
DLI	73.5 (65.3–81.8)	69.6 (60.4–78.7)	70.6 (61.6–79.6)	86.6 (82.3–90.6)	69.7 (60.5–78.8)
SBI	63.6 (55.5–71.1)	64.2 (55.1–73.1)	62.7 (54.5–70.1)	82.4 (78.4–86.0)	60.4 (51.6–68.4)
FSI	78.5 (71.1–86.0)	77.5 (69.7–85.0)	78.6 (70.6–86.2)	88.4 (84.3–92.5)	77.7 (70.2–85.1)
DLI + SBI	72.9 (65.3–80.2)	68.3 (59.2–77.1)	69.3 (60.3–77.7)	86.8 (82.7–90.3)	68.0 (59.3–76.6)
SBI + FSI	76.7 (68.6–84.3)	74.5 (66.1–82.6)	75.8 (66.9–83.8)	87.9 (83.6–91.9)	74.8 (66.2–82.8)
FSI + DLI	81.7 (74.4–88.4)	79.7 (71.3–86.9)	82.0 (74.2–88.4)	90.8 (86.9–94.0)	80.4 (72.2–87.3)
DLI + SBI + FSI	85.1 (78.5–90.0)	85.3 (78.1–91.2)	83.9 (76.4–90.7)	91.9 (88.4–95.1)	84.1 (77.0–90.4)

DLI: diffuse light imaging; SBI: slit-beam imaging; FSI: fluorescein staining imaging.

**FIGURE 6:**
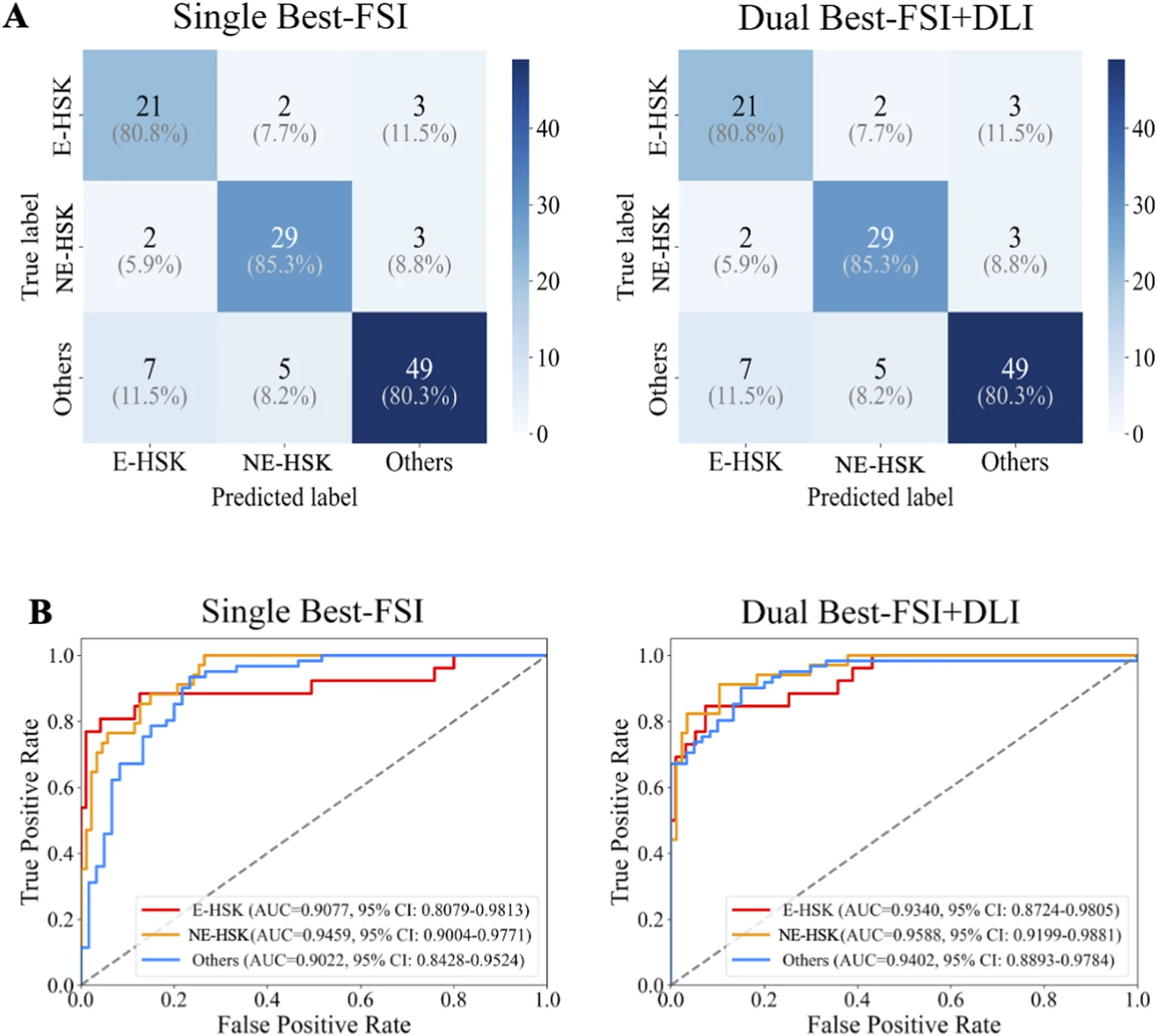
Best classification performance achieved by different numbers of modalities on the test dataset. **(A)** Best confusion matrices generated by single- and dual-modality approaches on the test dataset; **(B)** ROC curves generated for the corresponding image quality classification. E-HSK: epithelial herpes simplex keratitis; NE-HSK: Non-epithelial herpes simplex keratitis; Others: other corneal abnormalities; DLI: diffuse light imaging; SBI: slit-beam imaging; FSI: fluorescein staining imaging.

Pairing any two modalities consistently outperformed their individual counterparts, confirming the synergistic benefit of multimodal integration. Among all dual-modality combinations, FSI + DLI delivered the best performance, with an accuracy of 81.7% (95% CI: 74.4%–88.4%), a precision of 79.7%, a sensitivity of 82.0%, and a specificity of 90.8%. This synergy is clinically intuitive: FSI excels at highlighting superficial epithelial defects, while DLI clearly delineates global anterior-segment morphology, thereby compensating for the limitations of relying solely on global features or missing fine structural details.

The tri-modal fusion model achieved the highest diagnostic performance across all key metrics; compared to the best dual-modal baseline, the tri-modal architecture further improved accuracy from 81.7% to 85.1%. This peak performance indicates that the fusion layer successfully integrates global texture, depth contours, and local ulcer features into a unified high-dimensional manifold. This demonstrates that a comprehensive tri-modal approach is essential for robust, clinically applicable keratitis classification.

#### Heatmaps

To visualize the regions contributing most to the system, we generated a heatmap that superimposed a visualization layer at the end of the convolutional neural network (CNN). Grad-CAM technology is used to generate a “visual explanation” of the system’s decisions. This technique is applied to the final convolutional layer to generate a heatmap that highlights important regions in the input image. Brighter areas in the heatmap indicate regions that the network focuses on more during classification, suggesting that these areas contain more significant features. As shown in Figure 7, the model is able to effectively identify and focus on key image regions without being distracted by the background or other irrelevant information.

**FIGURE 7:**
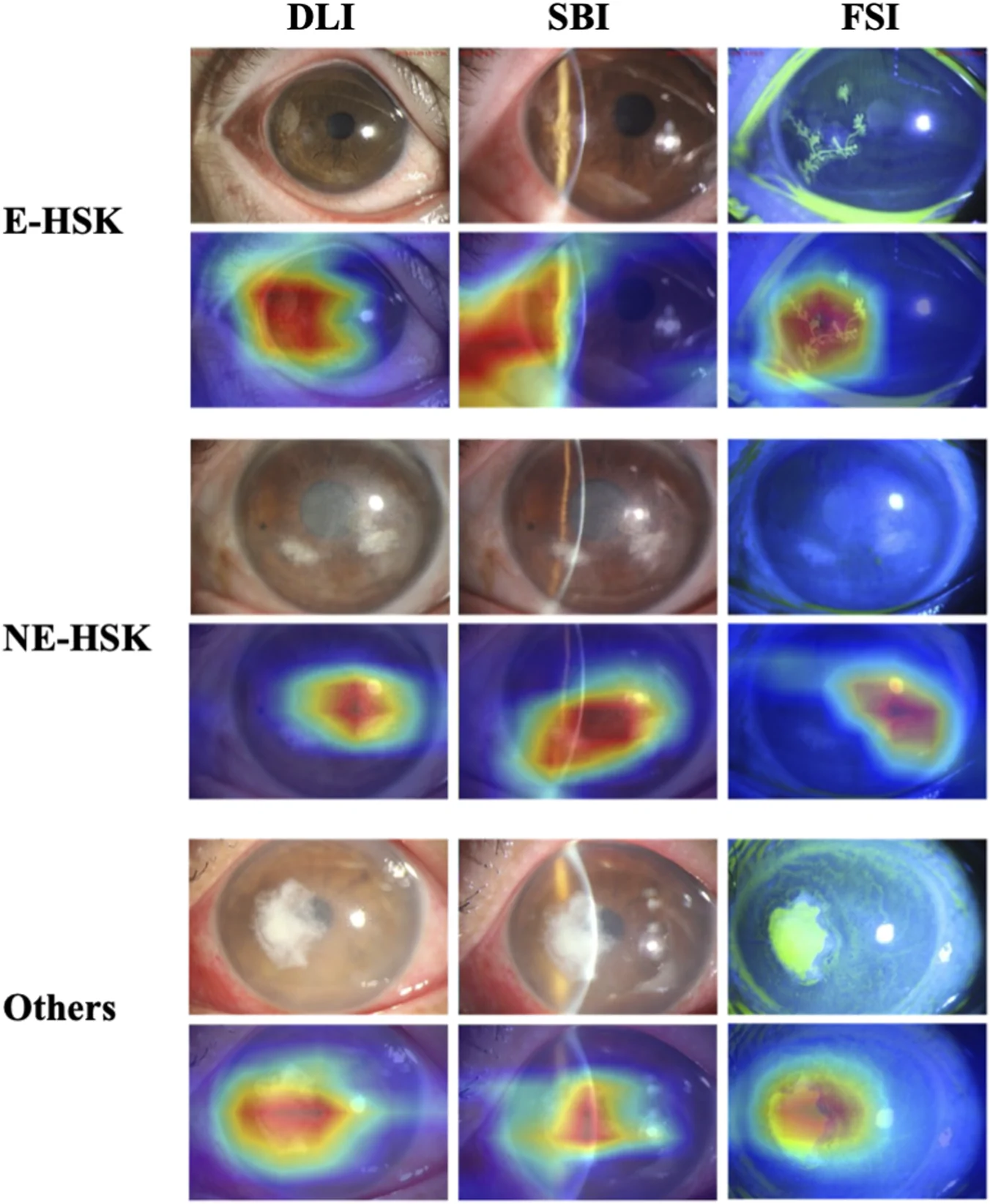
Grad-CAM heatmaps illustrating the model’s attention patterns across diagnostic categories and imaging modalities. Brighter regions denote areas of higher contribution to the classification output. E-HSK: epithelial herpes simplex keratitis; NE-HSK: Non-epithelial herpes simplex keratitis; Others: other corneal abnormalities; DLI: diffuse light imaging; SBI: slit-beam imaging; FSI: fluorescein staining imaging.

## Discussion

A critical unmet need in HSK management is a rapid, reliable tool for therapeutic subtyping—distinguishing E-HSK from NE-HSK. This distinction is clinically decisive: E-HSK requires antiviral monotherapy, whereas NE-HSK demands corticosteroid-based immunomodulation with antiviral cover. Misclassification can lead to severe outcomes, yet objective diagnostic guidance remains scarce, particularly in resource-limited settings. To address this, we developed DeepFusion-HSK, a multimodal DL framework integrating DLI, SBI, and FSI through a fusion architecture with second-order multiplicative interaction, dual-task learning, and dynamic gating. On internal testing, DeepFusion-HSK achieved 85.1% accuracy (95% CI: 78.5%–90.0%) and AUCs of 0.947, 0.949, and 0.940 for E-HSK, NE-HSK, and Others, outperforming Swin-Transformer (81.1%), EfficientNetV2 (77.6%), and ResNet50 (57.1%). This improvement arises from three design features: multiplicative fusion captures cross-modal pathological synergies, dual-task classification provides hierarchical contextual guidance, and dynamic gating mitigates softmax probability spillover. DeepFusion-HSK outperformed Swin-Transformer, a leading vision transformer, demonstrating that clinically informed multimodal integration and task-specific design can surpass generic model scaling for HSK subtyping. This finding aligns with the emerging consensus in ophthalmic AI that task-tailored multimodal approaches remain competitive with foundation models for well-defined clinical tasks.

A key strength of this study is the rigorous external validation on two independent datasets from distinct clinical centers, which is essential for assessing DL model generalizability in ophthalmology. External Dataset I (complete modalities) yielded 81.7% accuracy (95% CI: 75.4%–88.0%), a clinically acceptable decrease from internal performance (85.1%–81.7%). On External Dataset II (FSI absent), after light fine-tuning, accuracy reached 78.2% (95% CI: 70.3%–85.2%), only a 3.5% decrease, demonstrating robustness to missing modalities. This outperformance relative to unimodal baselines (3.2–6.0 percentage points on external sets) exceeds the typical 2%–7% gains reported for multimodal ophthalmic AI systems. Ablation studies showed a hierarchical contribution across modalities: FSI alone achieved 78.5%, DLI 73.5%, and SBI 63.6%. The lower standalone performance of SBI is attributable to the inherent properties of slit-beam imaging rather than the backbone: it captures only a narrow optical section, providing depth information but lacking the global morphology of DLI and the epithelial contrast of FSI; variations in slit position and angle across captures introduce slice alignment inconsistency; and signal concentration within a limited band lowers the effective signal-to-noise ratio. The FSI + DLI dual-modal combination reached 81.7%, while full tri-modal fusion further improved to 85.1%. This hierarchy mirrors clinical reasoning—DLI first for global morphology, SBI for depth assessment, and FSI for epithelial defect delineation—and confirms that each modality adds incremental value, with SBI contributing modest but meaningful edge-case discriminative power. Grad-CAM heatmaps confirmed that DeepFusion-HSK attends to clinically relevant regions, enhancing interpretability and clinical trust.

Fusion strategy affects real-world deployability beyond ideal-condition performance. In routine practice, individual modalities are often unavailable: fluorescein may be omitted, slit-beam skipped in busy clinics, or image quality compromised. Early- and intermediate-fusion architectures, which rely on complete modality alignment, are inherently brittle: any missing modality severely degrades or disables the model. Our fusion paradigm, by contrast, processes each modality independently and integrates at the feature level, conferring inherent resilience to incomplete inputs. The absence of one or even two modalities result in graceful degradation rather than complete failure. This was empirically demonstrated on Dataset II, where complete FSI dropout, after light fine-tuning, reduced accuracy only modestly from 81.7% to 78.2%, within acceptable clinical limits. This robustness makes DeepFusion-HSK adaptable to heterogeneous real-world data and supports its translation across diverse clinical settings.

Multimodal AI integrates heterogeneous data sources to provide a more comprehensive disease representation, improving diagnostic accuracy and prognostication (Muse and Topol, 2024; Wang et al., 2024; Tripathi et al., 2025). Image–clinical multimodal integration primarily involves two paradigms: slit-lamp imaging combined with clinical data and laboratory tests, and retinal imaging integrated with clinical data (Campo-Beamud et al., 2026; Dong et al., 2025). The main advantage of this integration is the combination of complementary information from imaging phenotypes and clinical parameters, enabling a more holistic and nuanced disease representation. The slit-lamp image–clinical–laboratory combination has proven particularly effective in the differential diagnosis of keratitis, whereas the retinal image–clinical data combination has shown potential for screening diabetes-related complications (Jin et al., 2026).

Several studies have demonstrated the utility of DL for keratitis classification. Ghosh et al. distinguished fungal from bacterial keratitis using 2,167 slit-lamp images, achieving an AUC of 0.904, sensitivity of 0.77, and F1 of 0.83 (Ghosh et al., 2022). Gu et al. developed a DL model on 5,325 ocular surface images for four corneal disease types, with AUCs exceeding 0.910 for each (Gu et al., 2020). Xu et al. reported a sequential deep model for infectious keratitis, attaining 80% accuracy on 2,284 images (Xu et al., 2021). Koyama et al. applied a hybrid DL algorithm to 4,306 slit-lamp images for pathogen probability estimation in infectious keratitis (Koyama et al., 2021). By contrast, Prajna et al. found limited multimodal gain over vision-only models in differentiating bacterial from fungal keratitis (AUROC 0.81; accuracy 77%) (Prajna et al., 2024). Table 6 provides a detailed comparison. In contrast to that finding, our results show consistent and substantial multimodal improvement, likely because bacterial–fungal differentiation can be captured by slit-lamp images alone, whereas HSK subtyping—distinguishing epithelial from stromal involvement—requires complementary information across illumination modalities.

**TABLE 6:** Summary of representative studies on AI-Based keratitis classification.

Study (Year)	Dataset (source, size)	Modalities	Classification Task	Model	Reported performance
Ghosh et al. (2022)	2,167 images, 194 patients (single center)	DLI	FK, BK	VGG19 + ensemble	Sensitivity 0.77; AUPRC 0.904; F1 0.83
Gu et al. (2020)	5,325 images (single center)	DLI	Infectious keratitis, non-infectious keratitis, corneal dystrophy or degeneration, corneal and limbal neoplasm	Hierarchical DL network	AUC >0.910
Xu et al. (2021)	2,284 images, 867 patients (single center)	DLI	BK, FK, HSK, and the others	Sequential-level DL model	Accuracy 0.80
Koyama et al. (2021)	4,306 images, 362 patients (single center)	DLI	AK, BK, FK, and HSK	InceptionResNetV2+GBDT	Accuracy 0.88; AUC >0.90
Li et al. (2024)	23055 images, 10592 patients (12 centers)	DLI	AK, BK, FK, HSK, and non-infectious keratitis	Dual-task DenseNet121	AUC >0.96; kappa 0.773
Prajna et al. (2024)	599 patients (MADURAI dataset)	DLI + clinical data	BK, FK	EfficientNetB7 + multimodal ML	AUPRC 0.94; AUC 0.81; accuracy 0.77; F1 0.85
This study	3,501 images (807 eyes) internal; 2,957 images external	DLI + SBI + FSI	E-HSK, NE-HSK, and others	Fusion DenseNet121 with dual-task + gating	Accuracy 85.1% (internal); 81.7%/78.2% (external)

AUPRC, area under the precision-recall curve; AUC, area under the receiver operating characteristic curve; BK, bacterial keratitis; DLI, diffuse light imaging; DL, deep learning; FK, fungal keratitis; AK, amebic keratitis; FSI, fluorescein staining imaging; HSK, herpes simplex keratitis; IK, infectious keratitis; SBI, slit-beam imaging; E-HSK: epithelial herpes simplex keratitis; NE-HSK: Non-epithelial herpes simplex keratitis; Others: other corneal abnormalities.

This study makes three contributions to the AI-assisted keratitis literature. First, whereas prior DL systems for infectious keratitis have focused on broad etiological classification (Zhang et al., 2026), DeepFusion-HSK targets fine-grained HSK therapeutic subtyping. This distinction is clinically decisive: E-HSK requires antiviral monotherapy and contraindicates corticosteroids, whereas NE-HSK requires corticosteroid immunomodulation with antiviral cover. To our knowledge, this is the first multimodal DL approach for this subtyping task. Second, most existing keratitis AI models rely on single-modality imaging (Zhang et al., 2026; Li et al., 2021; Li et al., 2024). Our integration of three complementary slit-lamp modalities yields substantial performance gains, consistent with the broader finding that multimodal systems outperform unimodal counterparts in ophthalmic applications (Jin et al., 2026). Third, external validation on two independent datasets, including one with complete FSI dropout, demonstrates generalizability under both ideal and challenging imaging conditions, addressing a gap in the literature. Together, these findings underscore the clinical utility and generalizability of DeepFusion-HSK, even with incomplete imaging inputs.

Several limitations warrant consideration. First, the retrospective design is subject to selection bias and may not fully capture real-world diversity. Although rigorous quality control was applied, the reference standard relied on clinical records rather than prospective PCR confirmation. This is consistent with standard practice: HSK diagnosis is primarily clinical, molecular confirmation is not routinely obtained, and PCR sensitivity varies substantially across subtypes. Prospective molecular validation is needed; future studies should incorporate standardized multimodal acquisition with PCR testing of corneal scrapings or tear samples. Second, external validation was limited to two Chinese centers. Performance in other geographic, ethnic, or healthcare contexts remains unknown, and international validation would strengthen translational evidence. Third, the fine-tuning strategy for External Dataset II is pragmatic but does not replace models natively robust to missing modalities. Future work should explore modality-agnostic or imputation-based architectures that adapt to available inputs without fine-tuning.

DeepFusion-HSK provides a clinically grounded framework for AI-assisted HSK subtyping, addressing the need for rapid, therapeutically actionable discrimination between E-HSK and NE-HSK. Its cross-center generalizability and resilience to complete FSI dropout support real-world deployment across diverse ophthalmic settings. This work demonstrates that task-specific, interpretable multimodal systems can help ophthalmologists, particularly early-career practitioners in resource-limited settings, make timely, accurate treatment decisions and prevent vision-threatening errors.

## Data Availability

The datasets presented in this article are not readily available because the data that support the findings of this study are available from the corresponding authors upon reasonable request. Requests to access the datasets should be directed to Qi Dai, dq@mail.eye.ac.cn.
