# Supplementary material for: DeepFusion-HSK: a multimodal deep learning for subtype classification of herpes simplex keratitis with multicenter validation
Source: Front Cell Dev Biol. 2026 Oct 19;14:1978214. doi: 10.3389/fcell.2026.1978214 (PMC13724611; doi:10.3389/fcell.2026.1978214)
Supplement: Supplementary file 1 [file DataSheet1.docx]

Supplementary Material

# 1 Supplementary Data

Supplementary Results: Baseline Comparison with Standard DenseNet121

To isolate the contribution of the proposed fusion architecture, a standard DenseNet121 baseline was trained, validated, and tested using the same data splits, random seed, and hyperparameters as DeepFusion-HSK. The baseline concatenated the three modality features $F_{concat}=Concat(F_{dli},F_{sbi},F_{fsi}）\in R^{3072}$and passed them to a single-task classifier, without dual-task classification or cross-modal interaction. The baseline achieved 76.0% accuracy (68.6–83.5), 80.2% precision (73.0–86.5), 77.4% sensitivity (69.3–84.9), 88.4% specificity (84.7–92.1), and 76.2% F1-score (67.8–83.7).

# 2 Supplementary Tables

## Supplementary Table 1. Comparison of class-imbalance handling strategies for the proposed multimodal fusion model

| Loss Function | Accuracy (%) | Precision (%) | Sensitivity (%) | Specificity (%) | F1-score (%) |
| --- | --- | --- | --- | --- | --- |
| **DeepFusion-HSK** | **85.1 (78.5-90.0)** | **85.3 (78.1-91.2)** | **83.9 (76.4-90.7)** | **91.9 (88.4-95.1)** | **84.1 (77.0-90.4)** |
| Focal loss | 82.6 (76.0-89.3) | 82.2 (74.4-89.2) | 80.2 (72.1-87.8) | 90.3 (86.3-94.1) | 81.0 (72.7-88.2) |

**Supplementary Table 2. Augmented training image counts per class and augmentation strategy.**

| Class | Original | RandomRotate | HorizontalFlip | VerticalFlip | CoarseDropout | Total |
| --- | --- | --- | --- | --- | --- | --- |
| E-HSK | 507 | 507 | 507 | 507 | 507 | 2535 |
| NE-HSK | 705 | 705 | 705 | 705 | 705 | 3525 |
| Others | 1239 | 1239 | 1239 | 1239 | 1239 | 6195 |
